# Migration under Climate Change in Southern Africa: A Nexus Planning Perspective

**DOI:** 10.3390/su12114722

**Published:** 2020-06-09

**Authors:** Sylvester Mpandeli, Luxon Nhamo, Sithabile Hlahla, Dhesigen Naidoo, Stanley Liphadzi, Albert Thembinkosi Modi, Tafadzwanashe Mabhaudhi

**Affiliations:** 1 Water Research Commission (WRC), Lynnwood Manor, Pretoria 0081, South Africa; 2 School of Environmental Sciences, University of Venda, Thohoyandou 0950, South Africa; 3 Centre for Transformative Agricultural and Food Systems (CTAFS), School of Agricultural, Earth and Environmental Sciences, University of KwaZulu-Natal, Scottsville, Pietermaritzburg 3209, South Africa

**Keywords:** adaptation, resilience, climate change, sustainability, livelihoods, vulnerability

## Abstract

Population increase is exacerbating resource insecurities due to increased demand for already depleted resources. Coupled with climate change, they are the main drivers of both intra-(rural-urban and urban-urban) and inter-migration (from one country to the other). We carried out a systematic review of literature, focusing on available options to ensure water and food security, as well as improve the socio-economic environment, highlighting the drivers of migration in southern Africa. The aim was to develop informed adaptation strategies and build resilience in the advent of accelerated migration. We developed a migration conceptual framework based on the nexus between water, food and socio-economic interlinkages. Urban areas in southern Africa are under immense pressure to accommodate climate refugees from resource stressed rural areas, a situation that is impacting on agricultural production. Most urban areas are exceeding their ecological thresholds to support the built environment, causing some socio-ecological challenges. Nexus planning can inform adaptation planning on permissible migration that are aligned with regional goals such as regional integration, poverty reduction and improved livelihoods. This would also contribute to the region’s achievements of the Sustainable Development Goals. Furthermore, through the identification of synergies and trade-offs, nexus planning can inform regional adaptation strategies for positively managing migration leading to sustainable outcomes.

## 1 Introduction

Climatic and environmental changes, coupled with population changes and migration, and their intricate relationships with development, are among the most pressing challenges dominating global sustainability discourses [1,2]. These unrelenting challenges are aggravating the vulnerabilities of most developing countries as they lack resources to cope and adapt [3,4]. Monocentric and linear approaches in resource management are no longer viable as they often promote divergent strategies and policies as well as entrench silos, which often results in negative trade-offs. For example, although southern Africa is endowed with vast natural resources, the region still struggles to achieve water, energy and food security for all [5,6]. Although it is acknowledged that the challenges of resource insecurities generally emanate from sector-based or silo, strategies in resources management, there are other underlying drivers such as migration, population growth and rapid urbanization [7,8]. Sector based approaches often inadvertently result in inequalities, increased vulnerabilities, tensions among the major stakeholders and disproportionate resource distribution, often triggering migration or concentration of populations in urban areas [5,9,10]. These factors are the main drivers of both intra-(rural-urban and urban-urban) and inter-migration (from one country to the other) [2].

The increasing socio-economic and environmental changes, together with increased frequency and intensity of extreme weather events such as droughts, cyclones, floods, heatwaves, among others, are proving to be detrimental to development thus triggering migration [11]. Between 2008 and 2016, extreme weather events, alone, displaced over 24 million people worldwide [12,13]. In addition, estimates by the Internal Displacement Monitoring Center (IDMC) indicate that weather-related disasters will result in 13.9 million people either being displaced or losing their livelihoods annually [13]. Elsewhere, the International Organization for Migration (IOM) projects that globally by 2050, over 200 million people will become climate migrants; this represents a ten-fold increase relative to current figures [14]. This gloomy picture on migration suggests that by 2050, one in every forty-five people would have been displaced by climate induced disasters. Compared with other regions, southern Africa is regarded as both a climate and migration hotspot due to a host of socio-economic, climatic and political challenges [15]. Of all the identified drivers of migration, climate change is recognized as a major driver as it has made some places inhabitable [16,17].

The effects of climate change on human migration will vary among different regions depending on several factors such as environmental and socio-economic conditions, culture, lifestyle and social networks [18]. However, there is a general consensus among researchers that climate change will mostly affect countries and regions whose populations depend on agriculture as a primary livelihood activity; this is due to the impact of increasing temperature and declining rainfall on crop production and harvest yields [17,18]. For example, increased weather variability and extremes in sub-Saharan Africa have forced many people to leave their home countries [19], while in Pakistan and Indonesia, the increase in temperature and heat waves have increased long-term or permanent migration [18]. Climate change also impacts on migration indirectly via socio-economic shocks or increases in food prices [18], possibly driving migration. However, most of these forced migrations, which are themselves a coping strategy, are unplanned hence creating challenges for both sending and receiving regions.

Migration could be an adaptation strategy, particularly when it is planned and coordinated in an integrated manner and is part of everyone’s history as it contributed to building the societies we are all part of today [20,21]. However, it can be a burden if the receiving area is not prepared for the mass influx of people, in terms of stretching infrastructure such as schools, water reticulation and health facilities and may exacerbate socio-economic challenges related to unemployment, poverty, inequality, crime rates, among others. Even in the presence of successful strategies to mitigate and adapt to climatic change impacts, climate change is still projected to result in migration levels above the current levels [22]. However, as an anticipatory response to climate change, migration is an important form of preparedness to change [23], but only in the presence of strategies that facilitate voluntary movement of people and avoid displacement or forced migration [24].

As risk reduction strategies are prioritizing mitigation, adaptation and building resilience to climate change, these strategies should also consider migration as an adaptation option [25,26]. This can be achieved through qualitative system dynamics modeling or transformative approaches to understand the intricately connected drivers of migration [27]. In particular, transformative approaches such as nexus planning, circular economy and sustainable food systems are essential for developing scenarios, which present opportunities to mitigate and adapt to the impacts of a climate change in an integrated manner [5]. Modeling through nexus planning simplifies the understanding of complex interlinkages between socio-economic and environmental drivers of migration, which is essential for informing decisions and pathways towards sustainable migration. Nexus planning can provide a simplified synopsis of the realities of migration and assist in identifying challenges and opportunities to achieve sustainable outcomes for both sending and receiving regions [28]. Transformative approaches indicate priority areas for immediate intervention for informed sustainable development in both urban and rural areas [28,29]. This is particularly relevant for southern Africa because of its uneven distribution and transboundary nature of resources, similar climate induced challenges and disproportionate economic wealth among regional countries [5]. Nexus planning addresses these issues by promoting sustainable urbanization and creation of climate smart cities through climate-smart initiatives and emergency preparedness at regional level [5,30]. However, rural-urban migration is exerting pressure on already overstrained infrastructure in urban areas and impacting on agricultural production in rural areas [31–33].

The aim of the study, therefore, is to provide integrated interventional solutions that can make this a reality. The objectives of the study were to demonstrate the usefulness of nexus planning and modelling in managing trade-offs and synergies—and as a transformative approach that links migration and climate change and as a pathway to transform urban areas into centers of climate action and adaptation—while at the same time maintaining and stimulating the productivity of rural areas. This fosters a win–win strategy, which will allow for climate action, both mitigation and adaptation and address livelihoods, income and water, energy and food security challenges in rural areas, which are the primary drivers of rural-to-urban migration. If successful, nexus planning and modeling can assist planners and decision-makers at regional, national and sub-national levels on how to manage the impacts of climate change, increase the absorptive capacity of regions (countries and cities alike), make agricultural systems more resilient and mitigate forced migration. When well-planned and coordinated in an integrated manner, migration can be viewed as an adaptation strategy, as well as a means of socio-economic development and regional integration.

## 2 Methods

### 2.1 Literature Review

We firstly conducted a literature review on migration and displacement focusing on southern Africa. This included developing a database of relevant publications using search engines such as Web of Science, Scopus and Google Scholar. The following terms were used for the search; impact of migration on agriculture, migration as an adaptation strategy to climate change, migration trends, drivers of migration, migration and urbanization, nexus planning, among other terms, from which a database of published articles was developed. To cover a wide range of relevant publications, we complemented published articles with reports related to climate influence on migration, drivers of migration, reasons for migrating, environment and migration, future scenarios of migration and pathways of migration. In addition. literature describing the migration-environment-displacement dynamics from other regions was also assessed to aid in understanding how these dynamics apply to southern Africa.

We noted that migration was well-studied, as evidenced by the quantity of available relevant publications (over 80 publications). The dominant topics cover areas such as climate change as an influencer of migration, impact of migration and migration trends. Of note is the existence of organizations dedicated on migration issues such as the United Nations High Commission for Refugees (UNHCR), International Organization for Migration (IOM), Global Commission on International Migration (GCIM) and Internal Displacement Monitoring Center (IDMC). These organizations (IOM and IDMC) have considerable published literature on migration, both at national and international levels.

However, from the literature review, we noted that little has been done on the role of transformative planning, particularly nexus approaches, in mitigating migration, and in transforming migration into an adaptation strategy in the advent of resource depletion and degradation and socio-economic changes. Through transformative and systems planning, migration frameworks can be developed to inform policy and decision-making on migration trends and preparedness that the projected mass influx of people into urban areas may not end as a burden. This study, therefore, focuses on the role of the water-food-socio-economic nexus in providing evidence on the impact of migration and in developing pathways to ensure resource security, improve livelihoods, reduce poverty and hunger and promote regional integration in southern Africa. Although the focus was on southern Africa, the method is applicable in other regions, adjusting it to particular situations. The study is informed by the projected increase in climate refugees to about 200 million refugees by 2050 [14], an increase expected to exert further pressure on already depleted resources [34].

### 2.2 Methodological Framework

**Figure 1:**
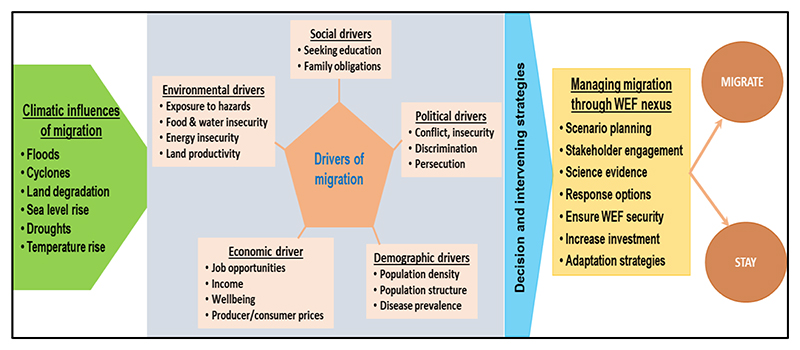
The methodological framework showing the key areas studied in the study. The framework highlights the Influence of socio-economic and environmental changes on migration.

The literature search assisted with identifying key thematic areas to direct the study, which included vulnerability, mitigation, preparedness, response and recovery. The thematic areas were linked with the main elements that drive migration which include water-food-socio-economic factors. Water-food-socio-economic elements formed the migration nexus that was used to develop a migration nexus conceptual framework. The approach simplified the understanding of the complex dynamics that are involved in the migration process. After identifying the thematic areas and linking them with nexus planning, we developed a thematic methodological framework (Figure 1) to guide the study. The methodological framework was used to assess the impacts of climate change and migration on resources. The conceptual framework provided a lens to aid in understanding the performance of resource planning, development and management in southern Africa—and how they are driving migration.

The study provides an understanding of the (a) drivers of migration in southern Africa and (b) intricate linkages between water-food-socio-economic in managing migration sustainably. The drivers of migration were grouped into five themes: social, political, economic, environmental and demographic [35,36] (Figure 1). An assumption was that the presence of spatial and temporal changes in one or more of these five themes creates the drivers of migration [37]. These themes and drivers interact or overlap differently across space, time and geography, hence can be adjusted to suit each region’s context. As already alluded, socio-economic, climate change and associated disasters are the major drivers of mass migration. Changes in climatic conditions in an area generally have a direct influence on migration, as climate induced disasters directly affect over 60% of the population of southern Africa who reside in rural areas, as they rely on natural systems for their livelihoods [28,38]. Climate change also has an influence on the economic drivers of migration such as employment opportunities, income, conflict and well-being [39,40].

The premise is that migration can become an adaptation strategy (not necessarily a challenge) if the receiving area has the resources and is prepared to absorb a high influx of migrants without shacking its systems. This can be achieved through integrated and transformative planning to manage present resources, but also mirroring into the future [41]. Nexus planning is particularly suited for integrated management and could be a pathway for regional integration, formulation of strategies that inform preparedness and proactive interventions [5].

## 3 Results and Discussion

### 3.1 Evidence from Literature: Migration Patterns, Risks and Impacts

On a continental scale, the number of migrants on the African continent increased from 15 million to 25 million between 2000 and 2017, representing an average increase of 2.8% per year, of which 47% are women. Major migration hubs are Abidjan in Ivory Coast, Johannesburg in South Africa and Nairobi in Kenya [41]. With regards to southern Africa, the region has recorded all types of migration that include mixed, irregular, labor and displacement [42]. Other causes include conflict, natural disasters, socio-economic inequalities and climate change [40,42]. In 2013, the region recorded over 4 million migrants (44% were female and 20% were under 19 years of age) [2]. Migration trends in the region indicate that South Africa absorbs the largest number of migrants (2.4 million) followed by the Democratic Republic of the Congo (447,000) and Zimbabwe (361,000) [2,43].

Southern Africa has also suffered the most from extreme weather events than the rest of the world [44–46], largely due to socio-economic and political challenges and the region’s high dependence on agriculture [19]. Climate change and its associated impacts have strong and direct impacts on agricultural production [19]. Climate induced events account for the largest percentage (67%) of natural disasters related deaths in the region [45]. Changes in rainfall patterns, cyclones and floods are negatively impacting on agriculture and regional water and food security as most countries rely on international aid to supplement food deficits [46]. The impacts of natural disasters on migration and human displacement have become significant both in scale and diverse in nature [45,47]. This is evidenced by the displacement of over 30% of pastoralists in East Africa due to climate change, and the percentage is projected to increase to about 40% by 2040 [48]. In sub-Saharan Africa, [19] project that anomalies in weather patterns could force 11.8 million people to migrate annually towards the end of the 21st century every year

Cyclonic flooding and severe drought are driving both internal and cross-border migration in the region [23]. Of all climate disasters, drought is recorded as the most widespread and devastating, as between 1900 and 2016, there were 702 recorded drought events worldwide and 312 of them occurred in Africa (Table 1) [46,47]. Since 1900 to-date, drought has caused about 900,000 deaths, and has affected over 414 million people on the African continent alone [47]. The total economic damage caused by drought is estimated at US$6.5 billion (Table 1). Thus, extreme weather events are predominantly responsible for most of the human displacement in southern Africa, apart from political and economic instability [49,50].

**Figure 2:**
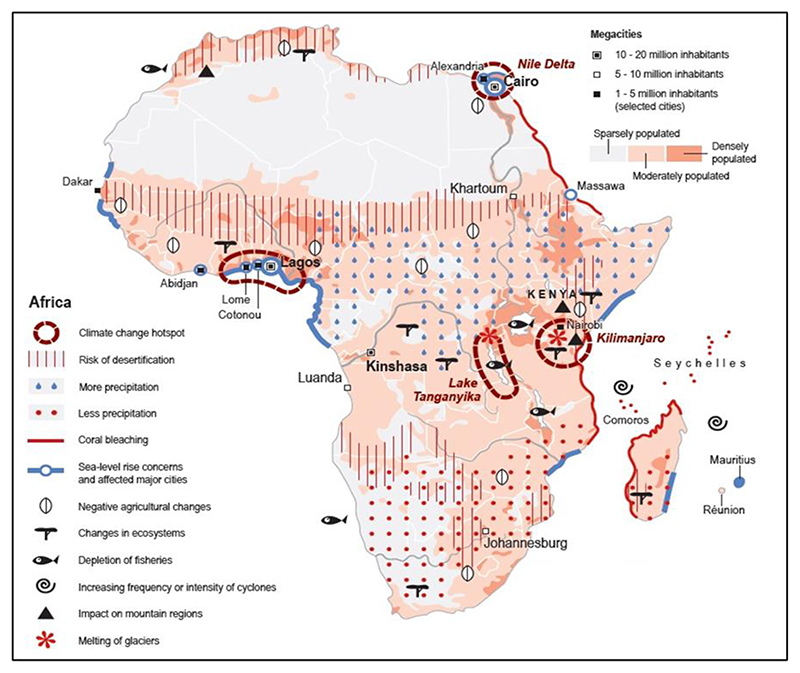
Key climatic risks and impacts (droughts, cyclones, desertification, wildfires); consequences (degradation of fisheries and loss of biodiversity, agriculture seasonal changes, depletion of water resources and limited ecosystem services); as well as socio-economic changes (vulnerable communities, major cities and densely populated regions prone to sea-level rise and other hazards) and their impacts on migration in Africa. The map also indicates climate change hotspots. **Source:** The Atlas of Environmental Migration, [51] (Ionesco et al., 2017).

The increasing risk of reduced precipitation, frequency and intensity of cyclones in the Atlantic coast of southern Africa—and the continued desertification, depletion of fisheries, environmental degradation, sea level rise and coral bleaching—over the coasts of Mozambique, Madagascar and Tanzania are driving migration in Africa (Figure 2). East Africa and the Great Lakes region are identified as climate change hotspots areas with increased melting of mountain glaciers and changes in ecosystems [51]. However, countries closer to the Equator are expected to receive above normal rainfall. Despite the negative impacts of climate change, population continue to increase, and the region will be moderately populated [51]. The combination of climate change and increasing population will also result in increased migration with cities like Johannesburg, Luanda and Kinshasa growing into megacities of more than 10 million people [51].

The risk of extreme weather events in Africa are worsening the already strained resources—and coupled with socio-economic and environmental changes—the continent needs to prepare for change and adapt for the future. These changes are undoubtedly affecting the already fragile resilience and adaptive strategies of the continent, requiring new approaches to address the challenges at hand.

### 3.2 Implications of Climate Refugees on Essential Resources

Southern Africa is endowed with fifteen resource rich transboundary river basins (IRBs), which present opportunities for regional integration through cross-sectoral coordination and management of resources. Management of resources at regional or transboundary level is a catalyst for sustained and inclusive economic growth that could ensure socio-economic security, as the resources are shared among countries [5,52]. Regional energy initiatives could be achieved through integrated cooperation of existing and planned renewable energy potential, and the agriculture potential could be achieved through cooperative flood management and irrigation development [52]. Cooperation among countries that share the Zambezi River Basin has potential to meet regional energy and irrigation requirements as it can generate some 30,000 Gigawatt hours (GWh)*/*year and unlock 774,000 ha of irrigated land [53]. However, despite such energy potential, biomass remains the main source of energy as only 24% of the total population and 5% of rural people have access to electricity [53]. Water resources are unevenly distributed as rainfall, oscillates between 100 and 2500 mm per annum [46]. Although agriculture potential is huge, crop production remains very low failing to meet the food requirements of a growing population [26]. Of the total land areas of about 9.9 million km^2^, 25% is arable and farming occurs only on 6% of the area [54,55].

The development of these resources at regional level has potential for the development of industrial nodes throughout the region [56]. This would provide opportunities for organized migration that translates into regional benefits for both the sending and receiving countries, particularly as an adaptation strategy.

### 3.3 Transforming Migration into a Climate Adaptation Strategy through Nexus Planning

Past experiences have shown that policies that are developed to restrict migration do not always succeed and are often counter-productive and self-defeating, as they exacerbate costs to the migrants, communities of origin and destination [57,58]. Nexus planning for climate-induced migration can provide decision support frameworks to guide policymakers on informed integrated migration policies, in the face of climate change. The approach has emerged as a transdisciplinary system for addressing polycentric and complex issues in an integrated manner, providing alternative pathways towards sustainable climate action and outcomes [29].

**Figure 3:**
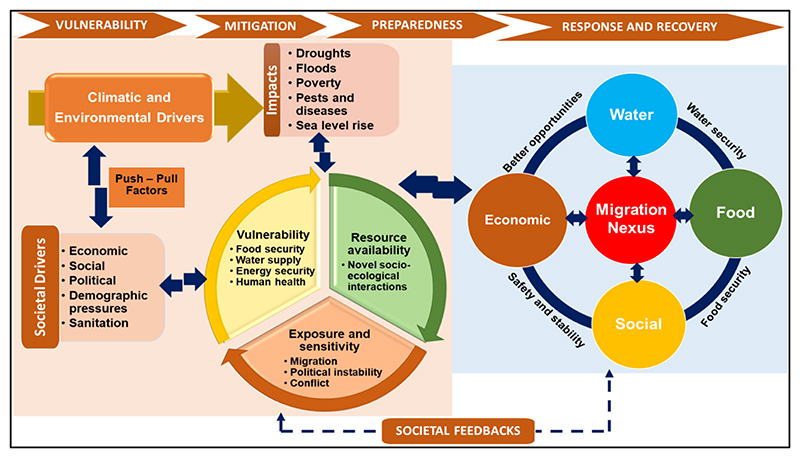
A water-food-socio-economic nexus conceptual framework indicating the drivers of migration and the factors involved in transforming migration into an adaptation strategy.

Migration becomes an adaptation strategy only when it is planned and people move from a high-risk place to a location where they are more secure from negative impacts [59]. This is a proactive approach as it is pre-planned and reduces risk and vulnerability. However, for this to happen in a sustainable manner, there is a need for evidence to inform integrated policies and decision-making. The migration nexus framework (Figure 3) illustrates the interlinkages and process within the water-food-socio-economic system and the drivers of migration. The migration nexus conceptual framework (Figure 3) illustrates the flow processes and nature of migration outcomes at a given point and time and space. Migration is influenced by societal, climatic and environmental changes, whose impacts results in either pull or push factors. The impacts determine the vulnerability of a place to change and inform whether to migrate due to resource availability, exposure and sensitivity. The migration nexus serves as a lens to understand the complex migration processes, simplifying the relationships between the components of the nexus that include water, food and socio-economic factors. The premise is to develop sustainable migration adaptation strategies and ensure inclusive region development and integration.

Nexus planning is oriented towards providing integrated solutions to complex systems through transformative and polycentric methods, other than linear or monocentric methods [28]. In developing the water-food-socio-economic nexus framework, we considered the complexity of integrating interlinked water and food security, climate action, social safety and stability and better opportunities and their heterogeneity over space and time and societal feedback (Figure 3); climate change was considered as a driver. Thus, nexus planning is preferred as it addresses complex relationships and their multi-causality within a nexus framework [60]. The approach provides pathways to achieve regional integration, respond to climate change, improve livelihoods, reduce poverty and offer better opportunities through sustainable migration. It simplifies the understanding of the critical drivers of change and factors that cause certain outcomes or the interactions that govern specific behaviors that exacerbate risk and vulnerability (Figure 3).

To achieve sustainable economic development at regional scale and ensure sustainable migration policies, a set of sustainability indicators related to water and food security, safety and stability and better opportunities are defined (Table 2). Sustainability indicators provide quantitative relationship among different components. The use of sustainability indicators in nexus planning is based on achieving sustainable development [61]. Sustainable development is meant to balance different and competing socio-ecological necessities [62]. A sustainable system provides for human needs and at the same maintaining a healthy environment [63,64]. Thus, sustainability indicators are simplified tools to unbundle the complex interrelationships among interdependent components of a system and turning those relationships into simple formulations that make assessments simpler [65]. The indicators given in Table 2 were derived from Sustainable Development Goals (SDGs) indicators and thus the approach can be used to assess progress towards SDGs.

**Figure 4:**
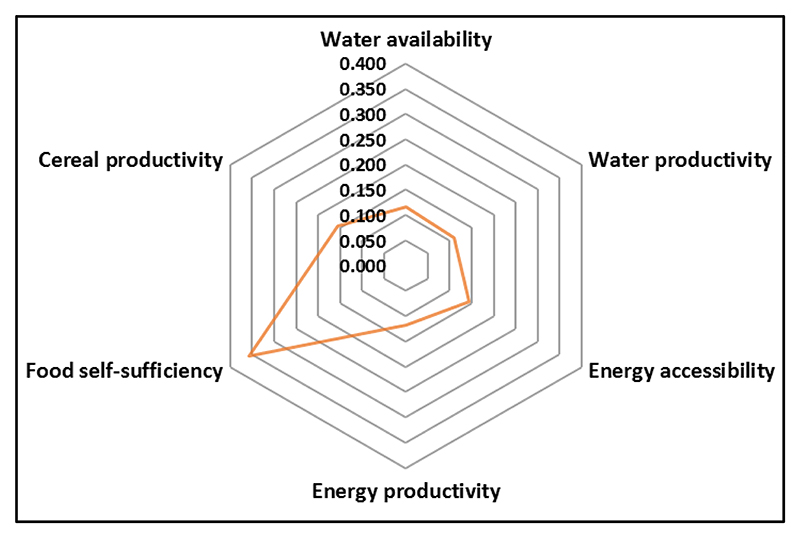
Current resources management in southern Africa, resembling an imbalanced development. The irregular shape of the yellow centerpiece indicates an unsustainable resources development in the region, which often leads to migration. **Source**: Mabhaudhi et al., 2019 [28].

The sustainability indicators (Table 2) were used to establish numerical relationships between WEF nexus sectors through the Analytic Hierarchy Process (AHP), a multicriteria decision-making method (MCDM) [29]. Through the pairwise comparison matrix (PMC) method of the AHP, indices for each indicator were defined as described by Nhamo et al. [29]. The indices were used to develop the spider graph (Figure 4) that established integrated quantitative relationship among the indicators [28].

The deformed and irregular shape of the centerpiece of the spider graph (Figure 4) is an indication of a poorly managed resource base, which normally leads to unsustainable development, which then triggers migration. Nexus planning thus becomes important for establishing the numerical relationships among the indicators, to indicate areas for priority intervention. In southern Africa, there is much focus on food self-sufficiency at the expense of other resources. The region needs to equally develop other sectors, achieve a circular shape of the centerpiece and create an environment for planned migration.

Nexus planning facilitates an integrated analysis of closely linked elements determining migration and the societal feedback (Figure 3). The key point is to manage climate-induced migration and gain mutual benefits in both receiving and sending areas. As a resilient and adaptive management approach, nexus planning for migration informs decisions in the face of uncertainty and unpredictable feedback and reduces risk and vulnerability that is caused by unpreparedness that may be caused by migration. Thus, the migration as an adaptation strategy focuses on thematic areas of vulnerability, mitigation, preparedness, response and recovery. Nexus planning in migration can reduce population pressure and exposure in high risk areas, while increasing the absorptive capacity of receiving areas. In terms of economic implications, mobility can represent an income diversification strategy, and it can play a key role in poverty reduction, mainly due to the remittances that are used for long-term investments in health and education opportunities. Therefore, nexus planning for climate-induced migration is win–win strategy which allows for climate action, including mitigation and adaptation, and the solving of numerous socio-economic challenges that southern Africa is currently facing. The migration nexus is thus a decision support framework that provides evidence-based intervention strategies to transform migration into a mitigation and adaptation strategy.

## 4 Recommendations

Southern Africa is facing several challenges that include climate change, rural to urban migration, population growth, food and nutrition insecurity, excessive water withdrawal, land degradation and deforestation, anthropogenic gas emissions, among others. These challenges are the major drivers of migration in southern Africa, and they require transformative approaches like nexus planning to transform migration into a mitigation and adaptation strategy. Nexus planning has the potential to reduce economic risk and enhances the mutual benefits of migration in both receiving and sending areas. There is need of relevant and coherent policies that link the adaptive strategies of migration in migrant communities to labor deficits in host nations. Circular migration schemes are a better option as they to allow seasonal labor migration. The challenge is regional and thus, requires regional approaches and strategies to turn migration into an adaptation strategy.

Despite the presence of regional legal and institutional frameworks, there is little evidence of their implementation to reduce the risk of migration [14]. The Southern African Development Community (SADC) has ratified the following regional legal frameworks on migration, but with little implementation: (i) SADC Protocol on Employment and Labor, (ii) Regional Labor and Migration Policy, which was adopted in 2014, (iii) Labor Migration Action Plan 2013–2015, (iv) Labor Migration Action Plan 2016–2019, (v) Draft Protocol on the Facilitation of Movement of Persons, adopted in 2005 and (vi) The Decent Work Program 2013–2019, adopted in 2013 [66,67]. There is a need to actively find solutions to climate migration as it threatens to cause devastating consequences on development, livelihoods and adaptation. However, focus was on political and economic migration, leaving behind climate migration.

Migration should no longer be viewed as a problem, but as an adaptation strategy as well as a policy for economic development. In this regard, migration can translate into economic growth through increased income tax revenues and skilled labor to the receiving area, especially if it is planned migration. The United States of America (USA) and the European Union (EU) have benefited from planned migration [68]. The bi-dimensional solutions of migration include addressing the drivers of migration in places of origin and formulating adaptation strategies that prepare the receiving place to accommodate migrants. Addressing the drivers of migration, should be accompanied by the enhancing the resilience in both receiving and sending areas in a holistic manner. Nexus approaches are best suited for such planning, particularly in fast-changing environments in which we live. We propose the following action areas to achieve sustainability through migration:

- There are great opportunities to benefit from leveraging and enhancing the flow of intra-regional labor migrants in southern Africa through exchange of scarce skills. Free movement of skilled labor in the region presents opportunities for inclusive economic development and regional integration, especially taking a cue from the USA and the EU;
- Planned migration presents triple benefits as the migrants themselves, the communities from where migrants come from, as well as the receiving area benefit economically [69]. This positive perspective does not consider migration as a burden on destination areas or lost brain drain on sending region;
- Coordinated regional migration has potential to enhance development strategies and could be poised to witness regional development nodes or industrial belts that could create employment opportunities through resource sharing as most of resources in the region are shared by countries [5];
- The region needs to collect disaggregated migration data in a systematic way, which may be used to inform policy decisions on addressing the drivers of migration through nexus planning. This will enable the formulation of context-based migration adaptation strategies. The analysis of such data provides long-term solutions to the challenges posed by migratory patterns such as demographic transitions and structural transformation in the society as driven by climate change.

## 5 Conclusions

Current trends and indications point to migration and other global challenges like climate change and rapid urbanization shaping the future of the world. Migration is projected to become more widespread and prevalent due to worsening impacts of and vulnerability to, environmental stressors. This study development a water-food-socio-economic nexus conceptual framework to illustrate the drivers of migration and the factors involved in transforming migration into an adaptation strategy. We also used a nexus planning model to establish numerical relationships among migration indicators to identify priority areas for intervention. This is relevant given the impact of climate change on agriculture-dependent livelihoods and resulting migration. Increasing temperatures have contributed towards a reduction in crop yields in the rainfed agricultural sector, reducing the contribution of agriculture to nations’ gross domestic products. This has already resulted in loss of livelihood and incomes for many, food and nutrition insecurity and health impacts, forcing people to move. Climate-induced migration is increasing pressure on already depleted water and energy resources in urban areas. Incorporating the migration nexus and nexus planning into developmental plans could go a long way in building resilience and adapting to future changes, resource insecurity and instability in southern Africa. Nexus planning has the potential to promote equitable development and resource distribution and improve the livelihoods of marginalized urban and rural communities, thereby building their resilience. Improving the response capacity and resilience of these communities could also provide incentive for migrants to stay in their homes and potentially decrease the rate of intra- and inter-migration.

## Figures and Tables

**Table 1:** Global drought frequency and impacts during 1900–2013.

Continent	Drought Events	People Killed	Affected People	Total Damage (‘000 US$)
**Africa**	312	867,131	414,235,329	6428,593
**Americas**	152	77	109,375,181	59,671,139
**Asia**	167	9663,400	2107,113,716	42,718,264
**Europe**	42	1200,002	15,488,769	25,481,309
**Oceania**	29	684	1074,2019	11,586,000
**Total**	702	11,731,294	2656,955,014	145,885,305

**Source**: EM-DAT: The International Disaster Database [47] (Guha-Sapir et al., 2019).

**Table 2:** Sustainability indicators to establish relationships among migration nexus planning. The indicators are the same with SDG indicators and thus, can be used to assess progress towards SDGs.

Component	Sub-Component	Indicator	SDG Indicator
Water	Water security	Proportion of available freshwater resources per capita	6.4.2
		Proportion of crops produced per unit of water used	6.4.1
Food	Food security	Prevalence of moderate/severe food insecurity in the population	2.1.2
		Proportion of sustainable agricultural production per unit area	2.4.1
Societal factors	Safety and stability	Conflict-related deaths per 100,000 population	16.1.2
		Proportion of population satisfied with public service experience	16.6.2
Economic factors	Better opportunities	Proportion of population living below the poverty line	1.2.1
		Proportion of population living in households with access to basic services	1.4.1
